# Effectiveness of a multidisciplinary heart failure disease management programme on 1-year mortality: Prospective cohort study

**DOI:** 10.1097/MD.0000000000004399

**Published:** 2016-09-16

**Authors:** Hervé Laborde-Castérot, Nelly Agrinier, Faiez Zannad, Alexandre Mebazaa, Patrick Rossignol, Nicolas Girerd, François Alla, Nathalie Thilly

**Affiliations:** a University of Lorraine, APEMAC EA4360; b Inserm U1116, CIC-P 1433, University Hospital of Nancy, Nancy; c F-CRIN INI-CRCT network; d Inserm U942; e University Paris Diderot, Sorbonne Paris Cité; f Department of Anesthesia and Critical Care, Hôpitaux Universitaires Saint-Louis Lariboisière, APHP, Paris; g Inserm CIC-EC 6, Clinical Epidemiology and Evaluation, University Hospital of Nancy, Nancy, France.

**Keywords:** disease management programme, heart failure, instrumental variable, observational study, propensity score

## Abstract

We performed a multicenter prospective observational cohort study (Epidémiologie et Pronostic de l’Insuffisance Cardiaque Aiguë en Lorraine, Epidemiology and Prognosis of Acute Heart Failure in Lorraine [EPICAL2]) to evaluate the effectiveness on mortality of a community-based multidisciplinary disease management programme (DMP) for heart failure (HF) patients.

Between October 2011 and October 2012, 1816 patients, who were hospitalized for acute HF or who developed acute HF during a hospitalization, were included from 21 hospitals in a northeast region of France. At hospital admission, their mean age was 77.3 (standard deviation [SD] 11.6) years and mean left ventricular ejection fraction was 45.0 (SD 16.0)%. A subset of patients were enrolled in a multidimensional DMP for HF (n = 312, 17.2%), based on structured patient education, home monitoring visits by HF-trained nurses, and automatic alerts triggered by significant clinical and biological changes to the patient. The DMP involved general practitioners, nurses, and cardiologists collaborating via an individual web-based medical electronic record. The outcome was all-cause mortality from the 3rd to the 12th month after discharge. During the follow-up, a total of 377 (20.8%) patients died: 321 (21.3%) in the control group and 56 (17.9%) in the DMP group. In a propensity score analysis, DMP was associated with lower 1-year all-cause mortality (hazard ratio 0.65, 95% CI 0.46–0.92). Instrumental variable analysis gave similar results (hazard ratio 0.56, 0.27–1.16).

In a real world setting, a multidimensional DMP for HF with structured patient education, home nurse monitoring, and appropriate physician alerts may improve survival when implemented after discharge from hospitalization due to worsening HF.

## Abstract 2

Supplemental Digital Content is available in the text

## 1 Introduction

Heart failure (HF) is a major public health problem, affecting approximately 1% to 2% of the adult population in developed countries, with the prevalence rising to ≥10% among people aged 70 years or more.^[1]^ Chronic HF is characterized by repeated hospitalizations and high mortality.^[2]^ A decrease in HF mortality has been observed in western countries over recent decades^[2,3]^ and probably relates to improvements in the management of HF, with the development of evidence-based therapies and clinical guidelines.^[4]^

Heart failure disease management programmes (HF-DMPs) are designed to improve outcomes through structured follow-up based on patient education, optimization of medical treatment, psychosocial support, and improved access to care.^[2]^ They are strongly recommended in HF guidelines to reduce the risk of HF hospitalization, based on the highest level of evidence.^[2]^ A Cochrane review of 11 randomized controlled trials concluded that case management interventions for HF were associated with a significant reduction in all-cause mortality at 12 months follow-up (odds ratio 0.66, 95% confidence interval [CI] 0.47–0.91).^[5]^ However, randomized controlled trials are generally conducted under ideal conditions, among selected patients being cared for by hyper-specialized physicians, none of which reflect real-world conditions. Accordingly, the generalizability of results from randomized controlled trials is open to question, particularly when the trials involve complex interventions such as HF-DMP, which are greatly context dependent.^[6]^ In addition, the magnitude of an intervention's effect under real-world conditions may be lower than in clinical trials. Thus, as a complement to trials, well-designed observational studies are useful to ascertain and quantify the effectiveness of HF-DMP in real-world settings.^[7]^

In this context, we used data from the Epidémiologie et Pronostic de l’Insuffisance Cardiaque Aiguë en Lorraine, Epidemiology and Prognosis of Acute Heart Failure in Lorraine (EPICAL2) cohort study to assess the effectiveness on all-cause 1-year mortality after hospitalization for acute heart failure (AHF), of a multidisciplinary community-based HF-DMP, implemented over several years in a large area of France. Our research hypothesis is that HF-DMP is an effective way to reduce mortality in a real-world setting, as demonstrated in randomized controlled trials.

## 2 Methods

### 2.1 Setting, design, and population

The EPICAL2 study was a prospective, observational community-based cohort study involving 21 volunteer hospitals spread over the Lorraine region of northeast France (population of 2,350,000, according to the 2012 census). The cohort enrolled comprised 2254 consecutive adult HF patients hospitalized between October 2011 and October 2012 in cardiology intensive care units, cardiology departments, or emergency departments at the hospitals concerned. Patients living in Lorraine and hospitalized for AHF were included, as were those who developed AHF during hospitalization. Eligible patients were identified either by physicians from the participating departments or by trained clinical research assistants who regularly visited the departments. Included patients were then followed for 3 years after discharge from the index hospitalization or until death if it happened first. The objectives of this cohort study were: to describe morbidity and mortality in the short-term (0–6 months) and mid-term (up to 3 years) and to identify the main prognostic factors; to assess the effectiveness of various aspects of care, in or out of hospital.

**Figure 1:**
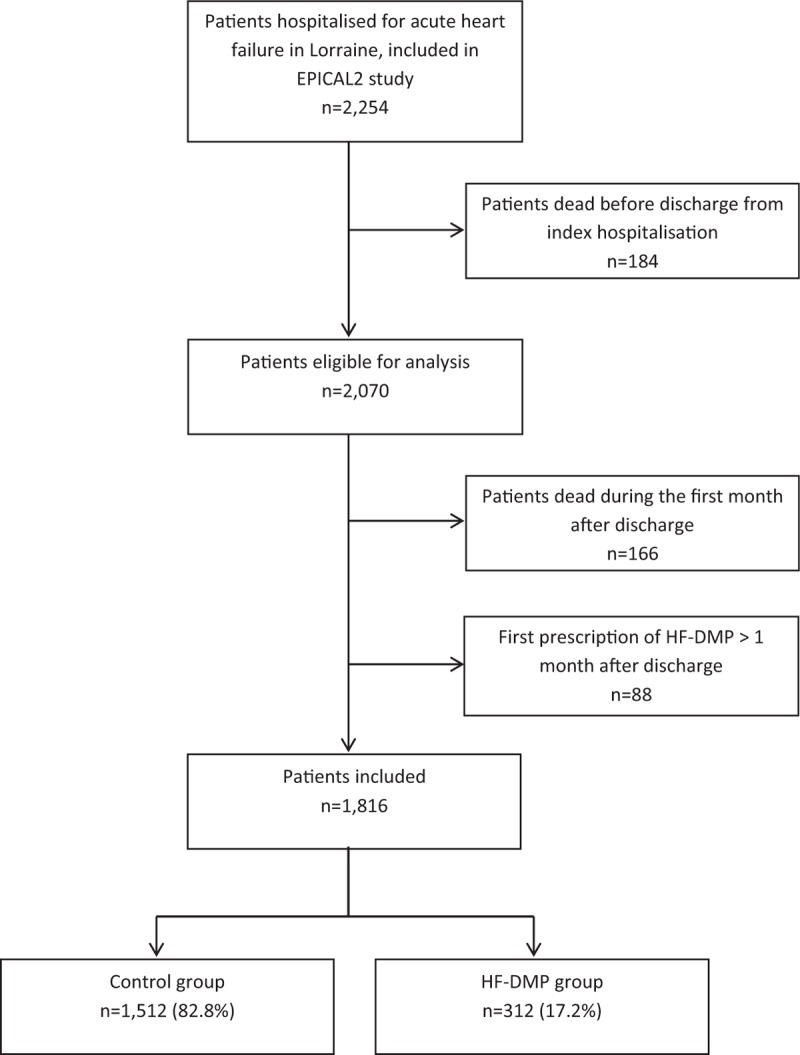
Flow chart for the selection of patients included in the evaluation of the heart failure disease management programme (HF-DMP).

In the present investigation, patients who died during the index hospitalization were excluded, leaving 2070 who were alive at hospital discharge (Fig. 1). Independently of EPICAL2, some of them may have been enrolled in routine care in an HF-DMP named Insuffisance CArdiaque en LORraine, Heart Failure in Lorraine (ICALOR), which was the only specialized DMP for HF patients implemented in Lorraine in 2011 to 2012. This HF-DMP was accessible to all HF patients living in the region, whatever the severity of their HF and ejection fraction. No specific inclusion (other than the HF diagnosis and area of residence) and exclusion criteria were established to be enrolled in ICALOR. The proposal to enroll a patient was left to physician discretion during hospitalization or outpatient care, and the patient was free to refuse it or to formally accept by signing a written consent. As an observational study, EPICAL2 did not affect the participation of patients in HF-DMP. The list of all HF-DMP enrolled patients and dates of consent were obtained from the ICALOR administrative database. A patient was considered as exposed to HF-DMP if he (she) signed the consent for HF-DMP enrollment before or during the index hospitalization, or during the 1st month after discharge, and had benefited from at least 1 part of the programme. For patients enrolled in an HF-DMP after discharge from index hospitalization, the time period between the cohort enrollment and the HF-DMP consent is “immortal,” as patients must survive this period in order to be exposed to HF-DMP.^[8]^ As recommended by Suissa,^[8]^ an adapted strategy was then implemented to control the immortal time bias: patients who died during the 1st month after hospital discharge and those who signed a consent to HF-DMP after the 1st month were excluded from this analysis. A total of 1816 patients were therefore considered in the present investigation (Fig. 1).

The EPICAL2 cohort study was conducted according to the principles of the Declaration of Helsinki and approved by national ethics committees (Comité Consultatif sur le Traitement de l’Information en Matière de Recherche, Comission Nationale de l’Informatique et des Libertés). All eligible patients were informed about the study protocol and were free to refuse to be included in the cohort.

### 2.2 The heart-failure disease management programme

ICALOR is a community-based HF-DMP 1st implemented in 2006 and independent of the EPICAL2 study described in detail elsewhere.^[9]^ Its main objectives were to reduce mortality and cardiology hospitalizations in HF patients. Briefly, in 2012, the programme involved 101 cardiologists, 926 general practitioners (GPs), and 1603 volunteer nurses collaborating in the home care of HF patients living in Lorraine and receiving the HF-DMP. After providing written consent, each patient's GP set up an individual web-based medical electronic record, accessible to all health professionals involved in ICALOR and the patient's care. Patients enrolled in ICALOR benefited first from a structured educational programme delivered by a dedicated team of trained nurses, and then received regular home visits from HF trained nurses who continued patient and family education and delivered information and counseling aimed at improving adherence to diet, medications, and patient self-care. The nurse intervention also focused on detection of worsening HF and managing comorbidities. At each home visit, nurses monitored blood pressure (BP), heart rate, and weight, and updated the patient's electronic records with routine laboratory results. The frequency of home-nurse visits were adapted to the clinical severity and evolution of the patient's condition. The ICALOR coordinating center received automatic alerts from the web-based electronic record system according to preset thresholds of variations in the following indicators: dyspnea, weight gain or loss, heart rate, edema, BP as monitored by the home-nurse, and B-type natriuretic peptide (BNP) or NT-proBNP, hemoglobin, potassium, sodium, and creatinine serum levels from laboratory assessments prescribed by the GP as a part of routine care. The coordinating center checked the validity of the alert and called the GP and/or the cardiologist to determine the appropriate action. The ICALOR HF-DMP included 304 (standard deviation [SD] 138) patients/year and triggered >500 alerts/year, more than half of them for weight gain.

### 2.3 Data collection and main outcome

A standardized form was used to interrogate medical records for socio-demographic and clinical data at inclusion, as well as biological and therapeutic data at inclusion, during the index hospitalization and discharge. In the present investigation, the main variables previously identified as impacting mortality in HF patients were considered as potential confounding factors: socio-demographic – age (<65, 65–79, ≥80 years), sex, living alone, type of hospital (local, regional, and teaching); clinical – HF etiology (ischemic or not), history of diabetes, chronic kidney disease, stroke or transient ischemic attack, chronic obstructive pulmonary disease or asthma, cancer, previous hospitalization for AHF, body mass index (BMI: underweight or normal weight < 25, overweight or obese ≥25 kg/m^2^), left ventricular ejection fraction (LVEF: <30, 30–44, ≥45%), low systolic BP (<115 mm Hg), and prolonged QRS duration on electrocardiogram (>120 ms); biological at admission – low glomerular filtration rate estimated by the MDRD equation^[10]^ (estimated glomerular filtration rate [eGFR]) (<60 mL/min/1.73 m^2^), hyponatremia (<135 mmol/L), hypokalemia (<3.8 mmol/L), anemia (hemoglobin < 10 g/dL), and BNP or NT-proBNP (BNP > 400 pg/mL or NT-proBNP > 450 pg/mL in patients <50 years old, NT-proBNP > 900 pg/mL in patients between 50 and 75 years old, NT-proBNP > 1800 pg/mL in patients >75 years old – according to the state of the art at the beginning of the cohort^[11]^).

All data were collected and checked for completeness, according to French Good Practices in Epidemiology,^[12]^ by 5 trained clinical assistants. Patient enrollment and quality of data collection were regularly controlled by a steering committee of 4 epidemiologists, 1 cardiologist, 1 nephrologist, and 1 cardiology intensive care physician. Ten percent of completed standardized forms were audited by an independent clinical research assistant who compared, for each form, data collected and the patient medical record.

### 2.4 Outcome of interest

The outcome of interest was all-cause mortality; the 1-year vital status of each patient and the date of death, if appropriate, were collected through civil registries. Survival time was calculated from the beginning of the 3rd month after discharge from the index hospitalization. This time zero for survival analysis was chosen because the 1st interventions of the HF-DMP might have been delayed by up to 1 month after patients gave their written consent. Surviving patients were censored at the end of the 1-year follow-up.

### 2.5 Statistical analysis

We first compared baseline characteristics of patients who received the HF-DMP with controls by calculating standardized differences (SDiffs), which indicate the degree of systematic differences in covariates between groups. Empirically, an absolute SDiff of <10% indicates a negligible difference in mean or percentage of the covariates between groups.^[13]^ Cox proportional hazards models were then used to assess the effect on 1-year mortality of HF-DMP. To minimize potential bias and confounding effects, a propensity score (PS) was estimated by using a multivariate logistic regression model including all patients’ baseline characteristics as shown in Table 1. The PS represents the likelihood of having received the HF-DMP depending on baseline characteristics of patients. The inverse probability of treatment weighting (IPTW) method was then applied by using the PS to assign individual weights to all observations, which allows some of characteristics of randomized controlled trials to be mimicked in observational study.^[14]^ Weights were stabilized by the marginal prevalence of the HF-DMP exposure.^[15]^ To check the accuracy of the PS model, we assessed covariate balance between groups after weighting by calculating SDiffs.^[16]^ Four Cox models were applied: model 1, PS analysis using stabilized IPTW; model 2, model 1 + adjustment for covariates for which SDiffs were >10% after IPTW; model 3, model 1 with trimming of 2.5% of patients (un)treated contrary to PS prediction on both sides;^[15]^ and model 4, model 2 with trimming of 2.5% of patients (un)treated contrary to PS prediction on both sides. Kaplan–Meier survival curves stratified by HF-DMP and control groups were constructed.

**Table 1:**
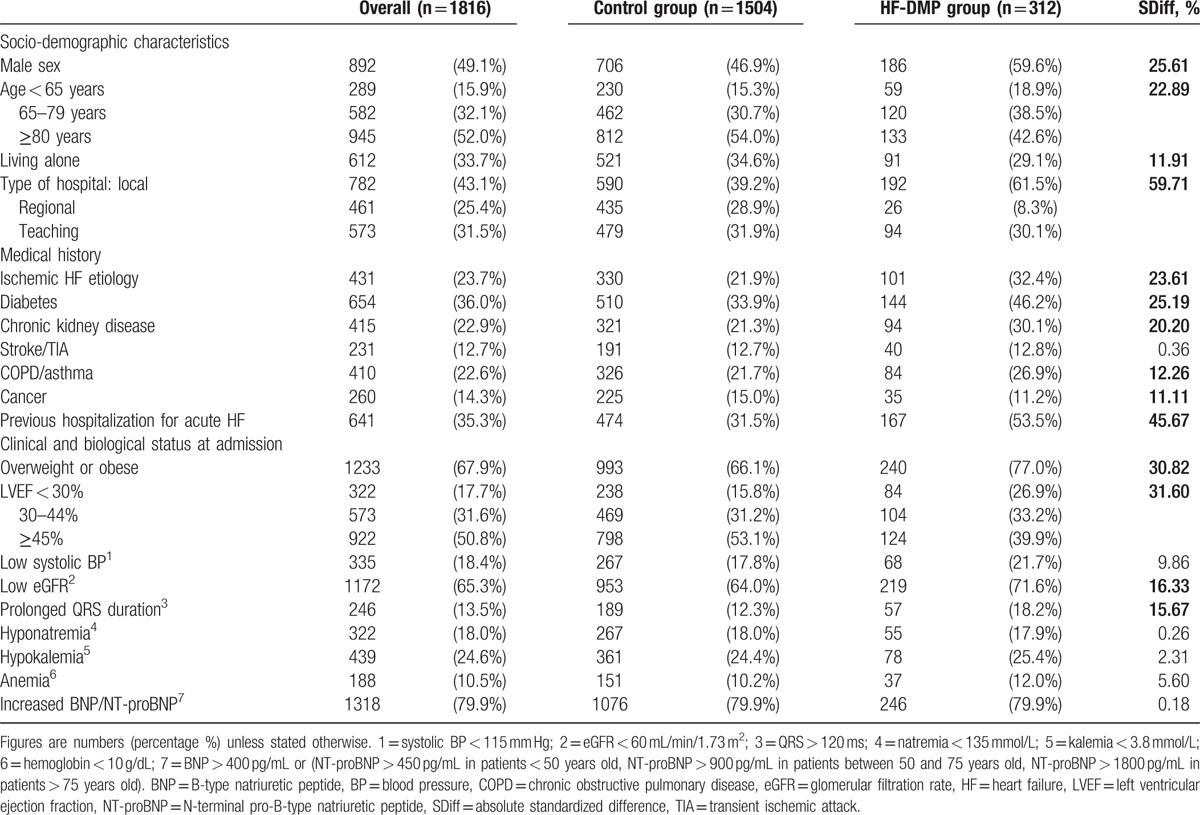
Baseline characteristics of included patients, overall, and according to heart failure disease management programme (HF-DMP) enrollment.

For 6 covariates with missing values (living alone, BMI, history of hospitalization for HF, systolic BP, LVEF, and prolonged QRS duration), we obtained values by multiple imputations as recommended for the Cox model analysis.^[17]^ This was achieved through regression switching imputation using linear or logistic regression models respectively for quantitative or qualitative incomplete covariate fitted. This procedure was repeated 5 times to obtain 5 draws for each missing value in 5 distinct datasets. Baseline characteristics of patients were described and compared in each dataset (see Supplemental content, Tables S1–S6. However, to be concise and summarize the results, Rubin approach was adopted, whereby the average percentage of each imputed covariate from the dataset of 5 is reported, the corresponding SDiff is calculated from these averages.^[18]^ To check the influence of multiple imputations on parameter estimates, a sensitivity analysis was conducted with the original nonimputed dataset.

The fit of the proportional hazards model was checked visually by plotting log (−log [survival]) versus log (time). Results are reported as pooled hazard ratios (HRs) with a 95% CI. All analyses were performed with SAS version 9.4 software (SAS Institute, Inc., Cary, NC).

### 2.6 Confirmatory analysis

To further assess the validity of our results, because of possible residual bias due to unmeasured confounders with the PS approach, we performed an additional instrumental variable (IV) analysis. This method requires a valid instrument which is strongly associated with the intervention (HF-DMP) (1st condition), does not directly affect mortality (2nd condition), and is not associated with confounding factors (3rd condition).^[19]^ A hospital's prescribing preference, defined as the prevalence of patients included in the HF-DMP in each hospital, appeared to be a good candidate. To test the association between the instrument and measured covariates, we classified hospitals into 2 groups according to their HF-DMP prescription rates (“DMP preference” group for hospitals above the median and “no-DMP preference” group for hospitals below the median) and compared characteristics of patients in both groups with absolute SDiffs. For the analysis, in the presence of a binary outcome and a binary exposure, we used 2-stage residual inclusion.^[20]^ The 1st-stage model used the IV and all observed covariates to predict the probability of receiving the HF-DMP. In the 2nd-stage, residuals from the 1st-stage were included as an additional variable along with the exposure and all observed covariates to model mortality in a Cox regression.

## 3 Results

### 3.1 Crude baseline characteristics

Overall, a total of 1816 patients were included in the present investigation (Table 1). The mean age was 77.3 (SD 11.6) years and 49.1% were men. The etiology of HF was ischemic for 23.7% of patients and the mean LVEF at hospital admission was 45.0 (SD 16.0)%. A total of 312 patients (17.2%) were enrolled in HF-DMP: 112 (35.9%) prior to index hospitalization, 90 (28.9%) during the index hospitalization, and 110 (35.3%) during the 1st month after discharge. In the HF-DMP group, patients received a median of 9 nurse home visits per year [Q1-Q3: 5–14] and triggered a median of 2.5 automatic alerts per year [Q1–Q3: 1–4]. Compared to controls, patients receiving HF-DMP were younger (mean age 74.7 [SD 11.2] years vs 77.8 [SD 11.6]), more likely to be men and hospitalized in a local hospital, and less likely to live alone. They also had more comorbidities (diabetes, chronic kidney disease, chronic obstructive pulmonary disease, overweight, or obesity), and were more likely to have ischemic HF. In addition, they had markers of high-risk HF as demonstrated by a higher frequency of a history of hospitalization for AHF and QRS enlargement along with lower LVEF, systolic BP, and eGFR.

The mean percentage of missing data among all 20 covariates considered was 4.5%; the highest proportions were for QRS duration (22.9%), BMI (19.7%), and LVEF (18.9%), whereas previous hospitalization for AHF, systolic BP, eGFR, natremia, kalemia, and anemia were missing less than 2% of data. Original nonimputed baseline characteristics of patients are available in Supplemental content (Table S1).

### 3.2 Baseline characteristics using IPTW and IV methods

Table 2 presents characteristics of patients in the 2 HF-DMP groups after using IPTW and IV methods. For both methods, covariates were well balanced between groups except for the type of hospital (SDiff = 15.05% for IPTW, SDiff = 137.64% for IV). In addition, SDiffs after IPTW were borderline for chronic kidney disease in average results (SDiff = 9.77%) and was slightly above 10% in 2 datasets (Supplemental content, Tables S5–S6). In the IV analysis, the proportion of patients enrolled in the HF-DMP was 32.9% (n = 222) in the “DMP preference” group and 9.2% (n = 105) in the “no-DMP preference” group (odds ratio 4.83 [3.71–6.31]).

**Table 2:**
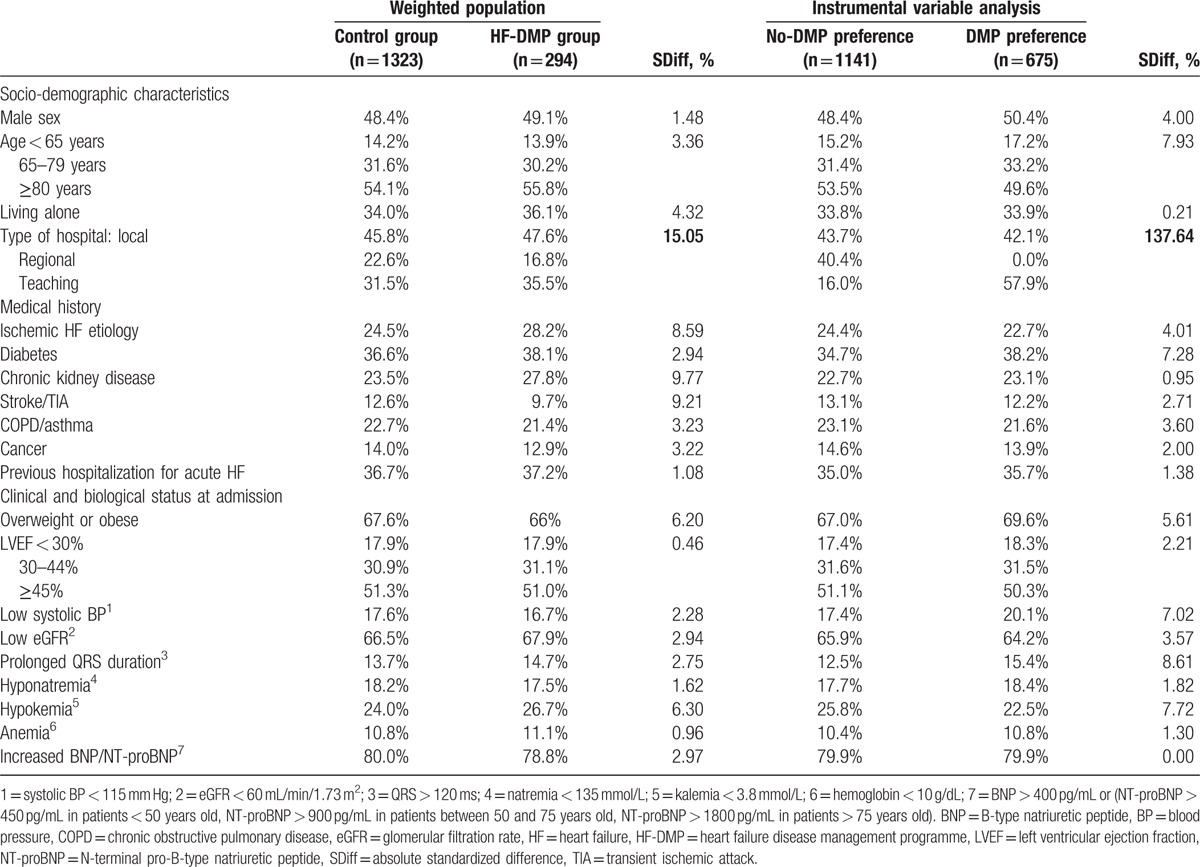
Characteristics of patients according to HF-DMP enrollment, after inverse probability of treatment weighting and in preference-based instrumental variable method.

### 3.3 Survival analysis

**Figure 2:**
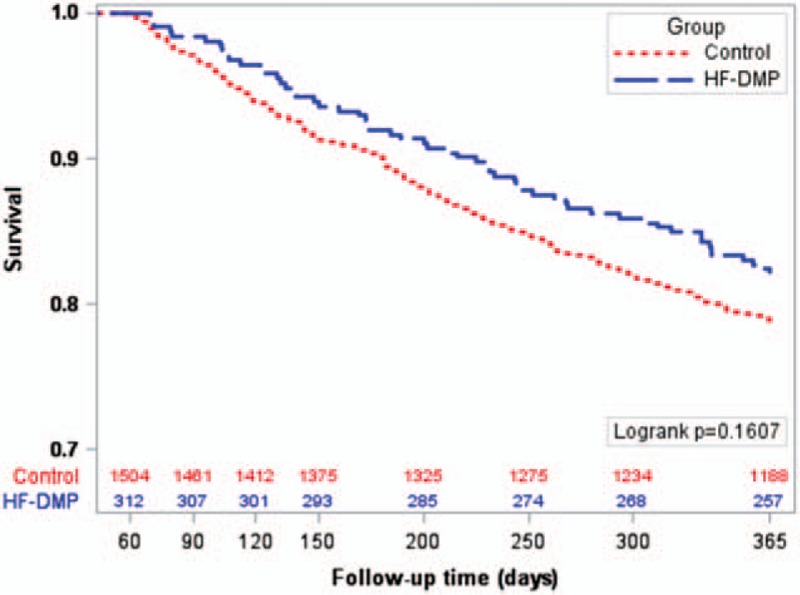
Kaplan–Meier survival curves according to the group (heart failure disease management programme [HF-DMP] and control). The numbers of patients still at risk of death are indicated.

Between the 3rd and the 12th months after the index hospitalization discharge, a total of 377 (20.8%) died, 321 (21.3%) controls, and 56 (17.9%) in the HF-DMP group (unadjusted Cox model HR 0.82, 95% CI 0.61–1.09). No patient was lost to follow-up. Kaplan–Meier survival curves of being dead according to the group (HF-DMP or control) show that mortality was higher in the control than the HF-DMP group, although the difference did not reach the statistical significance (Fig. 2).

Table 3 shows the estimations of the HF-DMP effect using the 4 Cox models. In the IPTW analysis performed in all included patients, HF-DMP was associated with significantly lower all-cause mortality (model 1 HR 0.65, 95% CI 0.46–0.92). Additional adjustment on the 2 covariates insufficiently balanced after IPTW (type of hospital and chronic kidney disease), provided similar intervention effect estimates (model 2 HR 0.63, 0.45–0.89). Slightly lower HRs were found when trimming the sample analyzed (model 3 HR 0.58, 0.40–0.86, and model 4 HR 0.58, 0.39–0.85). Multiple imputation provided similar point estimate as compared with analyses in nonimputed data and reduced estimation uncertainty (model 1 in nonimputed dataset HR 0.66, 0.38–1.12). IV analysis provided associations of similar strength to those observed in models 3 and 4 but did not reach statistical significance (HR 0.56, 0.27–1.16). The results of the survival analysis in the 5 datasets are available as Supplemental content (Table S7).

**Table 3:**
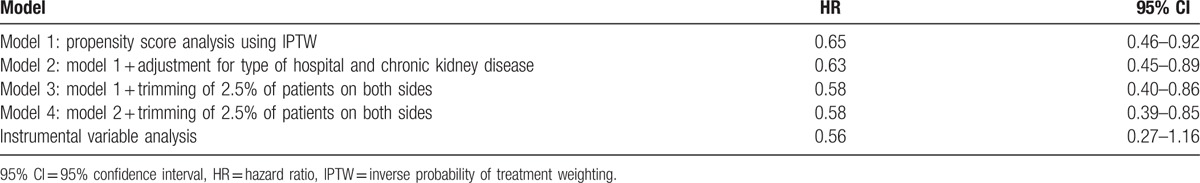
Estimations of the heart failure disease management programme effect.

## 4 Discussion

### 4.1 Principal results and possible explanations

Using a prospective cohort of 1816 patients with AHF hospitalized in Lorraine, we found evidence of real-world effectiveness of a multidisciplinary community-based HF-DMP in terms of all-cause 1-year mortality after hospitalization for HF. Several analytical methods (PS weighting, IV analysis) and models (with or without adjustments and/or trimming) were used to account for potentially confounding characteristics of patients that might affect their enrollment in HF-DMP. They all showed that patients enrolled in HF-DMP had a 35% to 44% reduced risk of death during the year after hospitalization, as compared to patients not enrolled. Our findings suggest that the efficacy of HF-DMP demonstrated in clinical trials may be translated into real-world clinical practice and further support the current guidelines recommending DMP a way of organizing care to improve outcome for HF patients.^[2]^ A DMP may include various health interventions with diverse design characteristics,^[21]^ and evaluations of such programmes should be interpreted in the light of type and characteristics of interventions considered. First, ICALOR interventions include patient education and training for caregivers aimed at enhancing patients’ adherence to medications, lifestyle, and dietary habits as well as medication prescription (i.e., according to guidelines), the final outcome being a reduced risk of decompensation of HF and then of HF hospitalization and death. Second, ICALOR interventions also include close home-monitoring for worsening signs and decompensation symptoms, which allows for prompt therapeutic adjustment at the beginning of HF aggravation. Here again, the objective is to avoid rehospitalization and, ultimately, death. In a previous ecological investigation, we found ICALOR to be associated with a reduction in HF hospitalizations in Lorraine, as compared to other regions of France.^[9]^ It could be hypothesized that the HF-DMP decreases the risk of HF hospitalizations, which leads to a lower likelihood of 1-year mortality. Last, ICALOR HF-DMP was implemented in Lorraine in 2006, that is, several years before the present investigation.^[9]^ It could therefore be assumed that years of experience of ICALOR resulted in a trained multidisciplinary network and contributed to the HF-DMPs effectiveness.

### 4.2 Comparisons with other studies

It has been shown that patients included in most clinical trials involving HF are not representative of the whole spectrum of HF patients actually managed in clinical practice,^[22]^ although clinical guidelines are based on results from such trials. This observation was confirmed in the present investigation, as patients enrolled in the ICALOR HF-DMP appeared to have more severe conditions than their peers included in clinical trials. For instance, the prevalence of eGFR below 60 mL/min/1.73 m^2^ observed in the present investigation was double that reported in a large (>1 million patients) meta-analysis assessing the impact of impaired kidney function in HF patients.^[23]^ Moreover, all 11 randomized controlled trials considered in the Cochrane meta-analysis reviewing the efficacy of case management interventions for HF on mortality excluded patients with comorbidities such as chronic kidney disease or those with a limited life expectancy, raising concern about the generalizability of their results.^[5]^ In addition, trials are generally conducted in tightly controlled conditions by research teams and involve strict protocols that maximize both intervention implementation and patient adherence.^[24]^ Consequently, the valuable contribution of the EPICAL2 cohort to existing evidence is related to its high external validity. To date, many observational studies have been conducted to assess the effectiveness of HF-DMP in relation to various outcomes, but those evaluating the HF-DMP effect on mortality in a prospective cohort design are scarce. To our knowledge only 2 observational prospective studies have published results concerning the efficacy of an HF-DMP including home nurse visits on all-cause mortality. The 1st, by Lowery et al,^[25]^ included 969 patients (458 [47%] in the HF-DMP group) and showed a significant 1-year all-cause mortality reduction in the HF-DMP group (HR 0.43, *P* < 0.001). However, this study was conducted in a very particular setting and applied specific exclusion criteria limiting the generalizability of the results to the whole spectrum of patients managed in clinical practice (patients were included in medical centers for veterans and had no comorbidity associated with a predicted life expectancy ≤6 months). The 2nd published study, by Bonarek-Hessamfar et al,^[26]^ included 362 patients (129 [36%] in HF-DMP group) with severe HF, with no indication for surgical or interventional treatment and no major disease reducing the short-term vital prognosis. After a 2-year follow-up period, the all-cause mortality was significantly lower in the HF-DMP group (HR 0.37, 0.16–0.89). However, analyses were adjusted for sex, age, and NYHA stage; the lack of control for many potential confounding factors foreshadowed a probable residual bias.

### 4.3 Strengths and limitations of our study

The main strengths of our study are the large cohort design, including an unselected sample of HF patients as encountered in current practice, the quality of individual data collection and the completeness of the 1-year follow-up, but also the use of analytical methods considered to be effective to control for confounders, in such an extensive manner that they are referred to as pseudo-randomized methods.^[27]^ The proposal of HF-DMP was left to the discretion of physicians and depended on their perception of the benefits to each patient of being enrolled, according to his (her) baseline characteristics. Thus, patients enrolled in ICALOR HF-DMP differed significantly in several ways from those not enrolled. These differences constituted a confounding by indication that usually threatens the internal validity of results from all observational studies.^[28]^ In our investigation, the risk was of underestimating the effect of HF-DMP, as patients enrolled in the HF-DMP had more severe HF than those who did not. To address the indication bias due the lack of random allocation of the HF-DMP, we used several analytical methods and models and obtained similar results, which tend to strengthen the credibility of our findings. At first we conducted a PS analysis using IPTW, which is considered by Austin^[29]^ to be the most effective PS method with which to reduce bias in observational studies dealing with time-to-event outcomes. After weighting on PS, patient baseline characteristics were well balanced between the groups (SDiff < 10%), except for the type of hospital and, marginally, for chronic kidney disease, but additional adjustment on these covariates did not impact on the result. IPTW analysis estimates the average effect of HF-DMP in the entire population and showed a significant 1-year mortality reduction in the HF-DMP group (model 1 HR 0.65, 0.46–0.92, and model 2 HR 0.63, 0.45–0.89). Slightly lower HRs were observed when excluding patients treated contrary to the prediction (model 3 HR 0.58, 0.40–0.86, and model 4 HR 0.58, 0.39–0.85), as recommended by Stürmer et al.^[15]^ PS analysis is recognized to be able to reduce bias due to all measured confounders, but fails to limit bias due to unmeasured or unknown confounders.^[15]^ To address this limit, IV analysis is increasingly used in clinical research, as it is able to control for measured confounders as well as unmeasured or unknown ones.^[19,27]^ In our investigation, hospital HF-DMP prescription preference appeared to be a good instrument for the analysis. By definition, hospital HF-DMP prescription rates are associated with HF-DMP (1st condition for IV use). Concerning the 2nd condition for IV use, hospital HF-DMP prescription preference can be assumed not to be associated with patient outcome (differences in the use of HF-DMP across hospitals are much more likely to be associated with nonmedical factors, such as local habits, rather than factors related to the HF itself). The 3rd condition for IV use, stipulating that the instrument must not be associated with confounding factors, was checked as patient characteristics were well balanced between “DMP preference” and “no-DMP preference” groups, except for 1 covariate, the type of hospital. This imbalance was explained by the presence of the only 2 regional hospitals in the “no-DMP preference” group. If regional and teaching hospitals were considered as one (i.e., large versus small care centers), the imbalance disappeared (data not shown). Finally, the IV analysis tends to confirm the 1-year mortality reduction associated with HF-DMP found in the PS analysis, even if the result did not reach statistical significance (HR 0.56, 0.27–1.16). As expected, IV analysis led to larger CIs than PS analysis because of a relatively limited sample size for the use of such a method but point estimate should be considered.^[30,31]^

Our results should be interpreted in the light of some limitations. First, eligible patients hospitalized in Lorraine during the study period were probably not all identified, and included in the EPICAL2 cohort. Identification of eligible patients based on declaration by physicians of the participating departments and/or by clinical research assistants was certainly not comprehensive. However, this type of recruitment also makes it unlikely that patients included in EPICAL2 were not representative of all eligible patients. In a previous study, the ICALOR HF-DMP coverage ratio, that is, the number of patients included in ICALOR over the number of HF patients requiring hospitalization in Lorraine was estimated at 18%, similar to the ratio that we found in the present investigation.^[9]^ The 2nd limitation was related to the rate of missing data in medical records for some covariates (i.e., QRS duration, BMI, and LVEF) which led us to use multiple imputation. However, a sensitivity analysis conducted with the original nonimputed dataset gave an HR similar to the analysis with imputed data, attesting to the lack of bias due to multiple imputation. The only difference is the nonsignificant result obtained with nonimputed data because of a loss of statistical power caused by the exclusion of patients with missing data. The 3rd limitation is related to some interesting questions that could not have been explored in the present investigation.

1. The limited number of events (377 deaths) in our study sample did not allow us to perform subgroups analyses such as analyses according to the LVEF level. Indeed, the question of the homogeneity of the HF-DMP effectiveness in reduced and preserved LVEF patients is particularly relevant as a large part of the HF-DMP activity is focused on optimizing and enhancing patient adherence to medications.
2. It would have been interesting to evaluate the effectiveness of the HF-DMP in terms of cardiac-specific mortality. However, cause of death was not collected and the choice of all-cause mortality as outcome is justified by the main objective of ICALOR to reduce overall mortality, whatever the cause.

The last limitation is the lack of some data that should have been considered in the PS because of their potential impact on prognosis, such as:

1. Biological results (especially BNP) and NYHA class collected at admission but unfortunately not discharge because these measurements are not systematically performed at discharge within routine clinical care in the hospitals involved in the EPICAL2 study and were not then available in medical records.
2. Remote monitoring, not collected in EPICAL2, is possible with some implantable cardiac devices and is likely to improve quality of care and hence survival. However, the small number of patients with such devices in our sample (n = 91, 5.0%) makes a noteworthy bias unlikely.

### 4.4 Conclusions and policy implications

This investigation shows that HF-DMP is likely to be effective in a real-world setting, with a reduction of 40% in 1-year mortality after hospitalization for HF patients enrolled in a community-based HF-DMP. Our results and those from previous randomized controlled and observational studies on HF-DMP provide strong evidence for a large decrease in HF mortality associated with HF-DMP in clinical practice. This finding is of critical importance given the low proportion of western populations that have access to HF-DMP.^[32,33]^ Health care systems in several countries do not provide HF-DMP at a national level or enforce the implementation of HF-DMP in all health care areas, resulting in moderate to poor coverage of HF-DMP across Europe.^[32]^ Given the high prevalence of HF and its poor prognosis, further promotion and development of community-based case management HF-DMP should significantly reduce the public health burden of HF.

## Supplementary Material

Supplemental Digital Content
