# Supplementary material for: Effectiveness of a multidisciplinary heart failure disease management programme on 1-year mortality: Prospective cohort study
Source: Medicine (Baltimore). 2016 Sep 16;95(37):e4399. doi: 10.1097/MD.0000000000004399 (PMC5402547; doi:10.1097/MD.0000000000004399)
Supplement: Supplemental Digital Content [file medi-95-e4399-s001.docx]

**Table S1.** Baseline characteristics of the patients according to heart failure disease management programme (HF-DMP) enrolment. Crude data without multiple imputation for missing data.

|  | Overall  (n=1816) | | Control group  (n=1504) | | HF-DMP group  (n=312) | | SDiff % |  |
| --- | --- | --- | --- | --- | --- | --- | --- | --- |
| *Socio-demographic characteristics* |  |  |  |  |  |  |  |  |
| Male sex | 892 | 49.1% | 706 | 46.9% | 186 | 59.6% | **25.61** |  |
| Age < 65 years | 289 | 15.9% | 230 | 15.3% | 59 | 18.9% | **22.89** |  |
| 65-79 years | 582 | 32.1% | 462 | 30.7% | 120 | 38.5% |  |  |
| ≥ 80 years | 945 | 52.0% | 812 | 54.0% | 133 | 42.6% |  |  |
| Living alone | 510 | 32.0% | 428 | 33.1% | 82 | 27.4% | **12.27** |  |
| Type of hospital: Local | 782 | 43.1% | 590 | 39.2% | 192 | 61.5% | **59.71** |  |
| Regional | 461 | 25.4% | 435 | 28.9% | 26 | 8.3% |  |  |
| Teaching | 573 | 31.5% | 479 | 31.9% | 94 | 30.1% |  |  |
| *Medical history* |  |  |  |  |  |  |  |  |
| Ischaemic HF aetiology | 431 | 23.7% | 330 | 21.9% | 101 | 32.4% | **23.61** |  |
| Diabetes | 654 | 36.0% | 510 | 33.9% | 144 | 46.2% | **25.19** |  |
| Chronic kidney disease | 415 | 22.9% | 321 | 21.3% | 94 | 30.1% | **20.20** |  |
| Stroke / TIA | 231 | 12.7% | 191 | 12.7% | 40 | 12.8% | 0.36 |  |
| COPD / asthma | 410 | 22.6% | 326 | 21.7% | 84 | 26.9% | **12.26** |  |
| Cancer | 260 | 14.3% | 225 | 15.0% | 35 | 11.2% | **11.11** |  |
| Previous hospitalisation for acute HF | 635 | 35.1% | 469 | 31.3% | 166 | 53.4% | **45.87** |  |
| *Clinical and biological status at admission* | |  |  |  |  |  |  |  |
| Overweight or obese | 994 | 68.2% | 595 | 66.3% | 202 | 78.3% | **34.01** |  |
| LVEF < 30% | 262 | 17.8% | 191 | 15.5% | 71 | 30.0% | **40.83** |  |
| 30-44% | 465 | 31.6% | 385 | 31.2% | 80 | 33.8% |  |  |
| ≥ 45% | 746 | 50.6% | 660 | 53.4% | 86 | 36.3% |  |  |
| Low systolic BP^1^ | 333 | 18.5% | 266 | 17.8% | 67 | 21.9% | **10.27** |  |
| Low eGFR^2^ | 1172 | 65.3% | 953 | 64.0% | 219 | 71.6% | **16.33** |  |
| Prolonged QRS duration^3^ | 172 | 12.3% | 127 | 11.0% | 45 | 18.4% | **21.17** |  |
| Hyponatraemia^4^ | 322 | 18.0% | 267 | 18.0% | 55 | 17.9% | 0.26 |  |
| Hypokalaemia^5^ | 439 | 24.6% | 361 | 24.4% | 78 | 25.4% | 2.31 |  |
| Anaemia^6^ | 188 | 10.5% | 151 | 10.2% | 37 | 12.0% | 5.60 |  |
| Increased BNP/NT-proBNP^7^ | 1318 | 79.9% | 1076 | 79.9% | 246 | 79.9% | 0.18 |  |

*Abbreviations.* BNP: B-type natriuretic peptide; BP: blood pressure; COPD: chronic obstructive pulmonary disease; eGFR: glomerular filtration rate; HF: heart failure; LVEF: left ventricular ejection fraction; NT-proBNP: N-terminal pro-B-type natriuretic peptide; SDiff: absolute standardized difference; TIA: transient ischaemic attack.
*Notes.* 1: systolic BP < 115 mmHg; 2: eGFR < 60mL/min/1.73m^2^; 3: QRS > 120 ms; 4: natraemia < 135 mmol/L; 5: kalaemia < 3.8 mmol/L; 6: haemoglobin < 10 g/dL; 7: BNP > 400 pg/mL or (NT-proBNP > 450 pg/mL in patients < 50 years old, NT-proBNP > 900 pg/mL in patients between 50 and 75 years old, NT-proBNP > 1800 pg/mL in patients > 75 years old)

**Table S2.** Characteristics of patients before and after inverse probability of treatment weighting and in preference-based instrumental variable approach in dataset 1.

|  |  | Unweighted population | |  |  | Weighted population | |  |  | Instrumental variable analysis | | |
| --- | --- | --- | --- | --- | --- | --- | --- | --- | --- | --- | --- | --- |
|  | Overall | Control group | HF-DMP group | SDiff % |  | Control group | HF-DMP group | SDiff % |  | No-DMP preference | DMP preference | SDiff % |
| N | 1816 | 1504 | 312 |  |  | 1326 | 297 |  |  | 1141 | 675 |  |
| *Socio-demographic characteristics* | | | | |  |  |  |  |  |  |  |  |
| Male sex |  | 46.9% | 59.6% | **25.61** |  | 48.4% | 49.3% | 1.77 |  | 48.4% | 50.4% | 3.99 |
| Age < 65 years |  | 15.3% | 18.9% | **22.89** |  | 14.1% | 14.0% | 3.67 |  | 15.2% | 17.2% | 8.00 |
| 65-79 years |  | 30.7% | 38.5% |  |  | 31.7% | 30.2% |  |  | 31.4% | 33.2% |  |
| ≥ 80 years |  | 54.0% | 42.6% |  |  | 54.1% | 55.8% |  |  | 53.5% | 49.6% |  |
| Living alone |  | 35.7% | 28.9% | **14.70** |  | 34.9% | 36.7% | 3.95 |  | 34.5% | 34.5% | 0.03 |
| Type of hospital: Local |  | 39.2% | 61.5% | **59.71** |  | 45.8% | 47.5% | **15.86** |  | 43.7% | 42.1% | **137.64** |
| Regional |  | 28.9% | 8.3% |  |  | 22.6% | 16.5% |  |  | 40.4% | 0.0% |  |
| Teaching |  | 31.9% | 30.1% |  |  | 31.6% | 36.0% |  |  | 16.0% | 57.9% |  |
| *Medical history* |  |  |  |  |  |  |  |  |  |  |  |  |
| Ischaemic HF aetiology |  | 21.9% | 32.4% | **23.61** |  | 24.5% | 27.9% | 7.69 |  | 24.4% | 22.7% | 4.00 |
| Diabetes |  | 33.9% | 46.2% | **25.19** |  | 36.7% | 37.9% | 2.58 |  | 34.7% | 38.2% | 7.31 |
| Chronic kidney disease |  | 21.3% | 30.1% | **20.20** |  | 23.5% | 27.2% | 8.41 |  | 22.7% | 23.1% | 0.98 |
| Stroke / TIA |  | 12.7% | 12.8% | 0.36 |  | 12.7% | 9.6% | 9.62 |  | 13.1% | 12.2% | 2.74 |
| COPD / asthma |  | 21.7% | 26.9% | **12.26** |  | 22.7% | 21.3% | 3.34 |  | 23.1% | 21.6% | 3.62 |
| Cancer |  | 15.0% | 11.2% | **11.11** |  | 14.0% | 12.8% | 3.46 |  | 14.6% | 13.9% | 1.78 |
| Previous hospitalisation for acute HF |  | 31.5% | 53.5% | **45.67** |  | 36.7% | 37.1% | 0.71 |  | 35.0% | 35.7% | 1.54 |
| *Clinical and biological status at admission* | | | |  |  |  |  |  |  |  |  |  |
| Overweight or obese |  | 65.8% | 77.9% | **32.90** |  | 67.5% | 65.4% | 7.51 |  | 66.6% | 69.9% | 7.34 |
| LVEF < 30% |  | 15.6% | 26.3% | **29.33** |  | 17.8% | 18.4% | 3.34 |  | 17.2% | 17.8% | 1.71 |
| 30-44% |  | 31.1% | 32.1% |  |  | 31.0% | 32.0% |  |  | 31.2% | 31.3% |  |
| ≥ 45% |  | 53.4% | 41.7% |  |  | 51.2% | 49.6% |  |  | 51.6% | 51.0% |  |
| Low systolic BP^1^ |  | 17.7% | 21.8% | **10.34** |  | 17.5% | 16.5% | 2.64 |  | 17.4% | 20.0% | 6.56 |
| Low eGFR^2^ |  | 64.0% | 71.6% | **16.33** |  | 66.5% | 67.5% | 2.18 |  | 65.9% | 64.2% | 3.48 |
| Prolonged QRS duration^3^ |  | 13.0% | 18.3% | **14.65** |  | 13.8% | 14.6% | 2.20 |  | 13.2% | 15.1% | 5.64 |
| Hyponatraemia^4^ |  | 18.0% | 17.9% | 0.26 |  | 18.1% | 17.4% | 1.76 |  | 17.7% | 18.4% | 1.83 |
| Hypokalaemia^5^ |  | 24.4% | 25.4% | 2.31 |  | 24.0% | 26.9% | 6.64 |  | 25.8% | 22.5% | 7.67 |
| Anaemia^6^ |  | 10.2% | 12.0% | 5.60 |  | 10.8% | 11.2% | 1.24 |  | 10.4% | 10.8% | 1.34 |
| Increased BNP/NT-proBNP^7^ |  | 79.9% | 79.9% | 0.18 |  | 80.0% | 79.1% | 2.22 |  | 79.9% | 79.9% | 0.09 |

*Abbreviations.* BNP: B-type natriuretic peptide; BP: blood pressure; COPD: chronic obstructive pulmonary disease; eGFR: glomerular filtration rate; HF: heart failure; LVEF: left ventricular ejection fraction; NT-proBNP: N-terminal pro-B-type natriuretic peptide; SDiff: absolute standardized difference; TIA: transient ischaemic attack.
*Notes.* 1: systolic BP < 115 mmHg; 2: eGFR < 60mL/min/1.73m^2^; 3: QRS > 120 ms; 4: natraemia < 135 mmol/L; 5: kalaemia < 3.8 mmol/L; 6: haemoglobin < 10 g/dL; 7: BNP > 400 pg/mL or (NT-proBNP > 450 pg/mL in patients < 50 years old, NT-proBNP > 900 pg/mL in patients between 50 and 75 years old, NT-proBNP > 1800 pg/mL in patients > 75 years old)

**Table S3.** Characteristics of patients before and after inverse probability of treatment weighting and in preference-based instrumental variable approach in dataset 2.

|  |  | Unweighted population | |  |  | Weighted population | |  |  | Instrumental variable analysis | | |
| --- | --- | --- | --- | --- | --- | --- | --- | --- | --- | --- | --- | --- |
|  | Overall | Control group | HF-DMP group | SDiff % |  | Control group | HF-DMP group | SDiff % |  | No-DMP preference | DMP preference | SDiff % |
| N | 1816 | 1504 | 312 |  |  | 1326 | 297 |  |  | 1141 | 675 |  |
| *Socio-demographic characteristics* | | | | |  |  |  |  |  |  |  |  |
| Male sex |  | 46.9% | 59.6% | **25.61** |  | 48.4% | 49.5% | 2.28 |  | 48.4% | 50.4% | 3.99 |
| Age < 65 years |  | 15.3% | 18.9% | **22.89** |  | 14.3% | 14.0% | 3.67 |  | 15.2% | 17.2% | 8.00 |
| 65-79 years |  | 30.7% | 38.5% |  |  | 31.6% | 30.1% |  |  | 31.4% | 33.2% |  |
| ≥ 80 years |  | 54.0% | 42.6% |  |  | 54.1% | 55.9% |  |  | 53.5% | 49.6% |  |
| Living alone |  | 34.5% | 28.9% | **12.20** |  | 33.9% | 35.5% | 3.36 |  | 33.9% | 32.9% | 2.18 |
| Type of hospital: Local |  | 39.2% | 61.5% | **59.71** |  | 45.8% | 48.3% | **15.41** |  | 43.7% | 42.1% | **137.64** |
| Regional |  | 28.9% | 8.3% |  |  | 22.6% | 16.5% |  |  | 40.4% | 0.0% |  |
| Teaching |  | 31.9% | 30.1% |  |  | 31.6% | 35.1% |  |  | 16.0% | 57.9% |  |
| *Medical history* |  |  |  |  |  |  |  |  |  |  |  |  |
| Ischaemic HF aetiology |  | 21.9% | 32.4% | **23.61** |  | 24.5% | 28.1% | 8.19 |  | 24.4% | 22.7% | 4.00 |
| Diabetes |  | 33.9% | 46.2% | **25.19** |  | 36.6% | 37.3% | 1.39 |  | 34.7% | 38.2% | 7.31 |
| Chronic kidney disease |  | 21.3% | 30.1% | **20.20** |  | 23.5% | 27.6% | 9.40 |  | 22.7% | 23.1% | 0.98 |
| Stroke / TIA |  | 12.7% | 12.8% | 0.36 |  | 12.6% | 9.8% | 8.77 |  | 13.1% | 12.2% | 2.74 |
| COPD / asthma |  | 21.7% | 26.9% | **12.26** |  | 22.7% | 21.6% | 2.65 |  | 23.1% | 21.6% | 3.62 |
| Cancer |  | 15.0% | 11.2% | **11.11** |  | 14.1% | 13.1% | 2.71 |  | 14.6% | 13.9% | 1.78 |
| Previous hospitalisation for acute HF |  | 31.5% | 53.5% | **45.67** |  | 36.7% | 37.0% | 0.59 |  | 35.1% | 35.7% | 1.35 |
| *Clinical and biological status at admission* | | | |  |  |  |  |  |  |  |  |  |
| Overweight or obese |  | 65.7% | 77.9% | **34.23** |  | 67.6% | 65.9% | 7.56 |  | 66.6% | 69.8% | 7.04 |
| LVEF < 30% |  | 16.0% | 26.6% | **32.53** |  | 17.8% | 18.1% | 0.63 |  | 17.6% | 18.1% | 2.13 |
| 30-44% |  | 30.5% | 34.3% |  |  | 30.4% | 30.3% |  |  | 31.5% | 30.5% |  |
| ≥ 45% |  | 53.6% | 39.1% |  |  | 51.8% | 51.6% |  |  | 50.9% | 51.4% |  |
| Low systolic BP^1^ |  | 17.8% | 21.5% | 9.21 |  | 17.5% | 16.6% | 2.37 |  | 17.4% | 20.2% | 6.93 |
| Low eGFR^2^ |  | 64.0% | 71.6% | **16.33** |  | 66.5% | 67.8% | 2.95 |  | 65.9% | 64.2% | 3.48 |
| Prolonged QRS duration^3^ |  | 13.0% | 16.7% | **10.43** |  | 13.6% | 14.2% | 1.65 |  | 12.3% | 15.9% | **10.32** |
| Hyponatraemia^4^ |  | 18.0% | 17.9% | 0.26 |  | 18.2% | 18.2% | 0.14 |  | 17.7% | 18.4% | 1.83 |
| Hypokalaemia^5^ |  | 24.4% | 25.4% | 2.31 |  | 23.9% | 26.8% | 6.69 |  | 25.8% | 22.5% | 7.67 |
| Anaemia^6^ |  | 10.2% | 12.0% | 5.60 |  | 10.8% | 11.0% | 0.61 |  | 10.4% | 10.8% | 1.34 |
| Increased BNP/NT-proBNP^7^ |  | 79.9% | 79.9% | 0.18 |  | 80.0% | 79.4% | 1.46 |  | 79.9% | 79.9% | 0.09 |

*Abbreviations.* BNP: B-type natriuretic peptide; BP: blood pressure; COPD: chronic obstructive pulmonary disease; eGFR: glomerular filtration rate; HF: heart failure; LVEF: left ventricular ejection fraction; NT-proBNP: N-terminal pro-B-type natriuretic peptide; SDiff: absolute standardized difference; TIA: transient ischaemic attack.
*Notes.* 1: systolic BP < 115 mmHg; 2: eGFR < 60mL/min/1.73m^2^; 3: QRS > 120 ms; 4: natraemia < 135 mmol/L; 5: kalaemia < 3.8 mmol/L; 6: haemoglobin < 10 g/dL; 7: BNP > 400 pg/mL or (NT-proBNP > 450 pg/mL in patients < 50 years old, NT-proBNP > 900 pg/mL in patients between 50 and 75 years old, NT-proBNP > 1800 pg/mL in patients > 75 years old)

**Table S4.** Characteristics of patients before and after inverse probability of treatment weighting and in preference-based instrumental variable approach in dataset 3.

|  |  | Unweighted population | |  |  | Weighted population | |  |  | Instrumental variable analysis | | |
| --- | --- | --- | --- | --- | --- | --- | --- | --- | --- | --- | --- | --- |
|  | Overall | Control group | HF-DMP group | SDiff % |  | Control group | HF-DMP group | SDiff % |  | No-DMP preference | DMP preference | SDiff % |
| N | 1816 | 1504 | 312 |  |  | 1326 | 297 |  |  | 1141 | 675 |  |
| *Socio-demographic characteristics* | | | | |  |  |  |  |  |  |  |  |
| Male sex |  | 46.9% | 59.6% | **25.61** |  | 48.3% | 48.8% | 1.02 |  | 48.4% | 50.4% | 3.99 |
| Age < 65 years |  | 15.3% | 18.9% | **22.89** |  | 14.3% | 13.9% | 3.13 |  | 15.2% | 17.2% | 8.00 |
| 65-79 years |  | 30.7% | 38.5% |  |  | 31.6% | 30.5% |  |  | 31.4% | 33.2% |  |
| ≥ 80 years |  | 54.0% | 42.6% |  |  | 54.1% | 55.7% |  |  | 53.5% | 49.6% |  |
| Living alone |  | 34.2% | 29.1% | 9.51 |  | 33.8% | 36.3% | 5.35 |  | 33.3% | 33.8% | 1.00 |
| Type of hospital: Local |  | 39.2% | 61.5% | **59.71** |  | 45.8% | 47.3% | **14.64** |  | 43.7% | 42.1% | **137.64** |
| Regional |  | 28.9% | 8.3% |  |  | 22.6% | 17.0% |  |  | 40.4% | 0.0% |  |
| Teaching |  | 31.9% | 30.1% |  |  | 31.6% | 35.7% |  |  | 16.0% | 57.9% |  |
| *Medical history* |  |  |  |  |  |  |  |  |  |  |  |  |
| Ischaemic HF aetiology |  | 21.9% | 32.4% | **23.61** |  | 24.4% | 28.0% | 8.28 |  | 24.4% | 22.7% | 4.00 |
| Diabetes |  | 33.9% | 46.2% | **25.19** |  | 36.7% | 38.6% | 4.04 |  | 34.7% | 38.2% | 7.31 |
| Chronic kidney disease |  | 21.3% | 30.1% | **20.20** |  | 23.5% | 27.8% | 9.85 |  | 22.7% | 23.1% | 0.98 |
| Stroke / TIA |  | 12.7% | 12.8% | 0.36 |  | 12.6% | 9.6% | 9.37 |  | 13.1% | 12.2% | 2.74 |
| COPD / asthma |  | 21.7% | 26.9% | **12.26** |  | 22.7% | 21.2% | 3.79 |  | 23.1% | 21.6% | 3.62 |
| Cancer |  | 15.0% | 11.2% | **11.11** |  | 14.0% | 12.7% | 3.76 |  | 14.6% | 13.9% | 1.78 |
| Previous hospitalisation for acute HF |  | 31.5% | 53.5% | **45.67** |  | 36.8% | 37.0% | 0.48 |  | 35.1% | 35.7% | 1.35 |
| *Clinical and biological status at admission* | | | |  |  |  |  |  |  |  |  |  |
| Overweight or obese |  | 65.6% | 76.3% | **29.00** |  | 67.1% | 66.5% | 2.89 |  | 66.5% | 68.9% | 5.37 |
| LVEF < 30% |  | 15.8% | 27.2% | **30.10** |  | 18.0% | 17.6% | 1.19 |  | 17.6% | 17.9% | 1.55 |
| 30-44% |  | 31.6% | 31.4% |  |  | 30.8% | 31.1% |  |  | 31.8% | 31.1% |  |
| ≥ 45% |  | 52.7% | 41.4% |  |  | 51.2% | 51.3% |  |  | 50.6% | 51.0% |  |
| Low systolic BP^1^ |  | 17.7% | 21.8% | **10.34** |  | 17.5% | 16.6% | 2.40 |  | 17.4% | 20.0% | 6.56 |
| Low eGFR^2^ |  | 64.0% | 71.6% | **16.33** |  | 66.5% | 68.0% | 3.21 |  | 65.9% | 64.2% | 3.48 |
| Prolonged QRS duration^3^ |  | 12.1% | 18.9% | **18.90** |  | 13.6% | 14.2% | 1.52 |  | 12.3% | 15.0% | 7.86 |
| Hyponatraemia^4^ |  | 18.0% | 17.9% | 0.26 |  | 18.2% | 17.1% | 2.92 |  | 17.7% | 18.4% | 1.83 |
| Hypokalaemia^5^ |  | 24.4% | 25.4% | 2.31 |  | 23.9% | 26.4% | 5.71 |  | 25.8% | 22.5% | 7.67 |
| Anaemia^6^ |  | 10.2% | 12.0% | 5.60 |  | 10.8% | 11.2% | 1.22 |  | 10.4% | 10.8% | 1.34 |
| Increased BNP/NT-proBNP^7^ |  | 79.9% | 79.9% | 0.18 |  | 80.0% | 77.9% | 5.14 |  | 79.9% | 79.9% | 0.09 |

*Abbreviations.* BNP: B-type natriuretic peptide; BP: blood pressure; COPD: chronic obstructive pulmonary disease; eGFR: glomerular filtration rate; HF: heart failure; LVEF: left ventricular ejection fraction; NT-proBNP: N-terminal pro-B-type natriuretic peptide; SDiff: absolute standardized difference; TIA: transient ischaemic attack.
*Notes.* 1: systolic BP < 115 mmHg; 2: eGFR < 60mL/min/1.73m^2^; 3: QRS > 120 ms; 4: natraemia < 135 mmol/L; 5: kalaemia < 3.8 mmol/L; 6: haemoglobin < 10 g/dL; 7: BNP > 400 pg/mL or (NT-proBNP > 450 pg/mL in patients < 50 years old, NT-proBNP > 900 pg/mL in patients between 50 and 75 years old, NT-proBNP > 1800 pg/mL in patients > 75 years old)

**Table S5.** Characteristics of patients before and after inverse probability of treatment weighting and in preference-based instrumental variable approach in dataset 4.

|  |  | Unweighted population | |  |  | Weighted population | |  |  | Instrumental variable analysis | | |
| --- | --- | --- | --- | --- | --- | --- | --- | --- | --- | --- | --- | --- |
|  | Overall | Control group | HF-DMP group | SDiff % |  | Control group | HF-DMP group | SDiff % |  | No-DMP preference | DMP preference | SDiff % |
| N | 1816 | 1504 | 312 |  |  | 1313 | 283 |  |  | 1141 | 675 |  |
| *Socio-demographic characteristics* | | | | |  |  |  |  |  |  |  |  |
| Male sex |  | 46.9% | 59.6% | **25.61** |  | 48.3% | 48.5% | 0.50 |  | 48.4% | 50.4% | 3.99 |
| Age < 65 years |  | 15.3% | 18.9% | **22.89** |  | 14.2% | 14.0% | 3.78 |  | 15.2% | 17.2% | 8.00 |
| 65-79 years |  | 30.7% | 38.5% |  |  | 31.6% | 30.1% |  |  | 31.4% | 33.2% |  |
| ≥ 80 years |  | 54.0% | 42.6% |  |  | 54.1% | 55.9% |  |  | 53.5% | 49.6% |  |
| Living alone |  | 35.2% | 30.1% | **10.77** |  | 34.6% | 36.6% | 4.22 |  | 34.2% | 34.5% | 0.71 |
| Type of hospital: Local |  | 39.2% | 61.5% | **59.71** |  | 45.9% | 47.7% | **13.88** |  | 43.7% | 42.1% | **137.64** |
| Regional |  | 28.9% | 8.3% |  |  | 22.6% | 17.2% |  |  | 40.4% | 0.0% |  |
| Teaching |  | 31.9% | 30.1% |  |  | 31.3% | 35.1% |  |  | 16.0% | 57.9% |  |
| *Medical history* |  |  |  |  |  |  |  |  |  |  |  |  |
| Ischaemic HF aetiology |  | 21.9% | 32.4% | **23.61** |  | 24.4% | 28.7% | 9.70 |  | 24.4% | 22.7% | 4.00 |
| Diabetes |  | 33.9% | 46.2% | **25.19** |  | 36.6% | 38.1 % | 3.15 |  | 34.7% | 38.2% | 7.31 |
| Chronic kidney disease |  | 21.3% | 30.1% | **20.20** |  | 23.6% | 28.3% | **10.85** |  | 22.7% | 23.1% | 0.98 |
| Stroke / TIA |  | 12.7% | 12.8% | 0.36 |  | 12.7% | 9.7% | 9.55 |  | 13.1% | 12.2% | 2.74 |
| COPD / asthma |  | 21.7% | 26.9% | **12.26** |  | 22.8% | 21.4% | 3.30 |  | 23.1% | 21.6% | 3.62 |
| Cancer |  | 15.0% | 11.2% | **11.11** |  | 14.0% | 12.8% | 3.71 |  | 14.6% | 13.9% | 1.78 |
| Previous hospitalisation for acute HF |  | 31.5% | 53.5% | **45.67** |  | 36.6% | 36.9% | 0.47 |  | 35.0% | 35.7% | 1.54 |
| *Clinical and biological status at admission* | | | |  |  |  |  |  |  |  |  |  |
| Overweight or obese |  | 66.9% | 76.9% | **28.59** |  | 68.1% | 65.4% | 8.10 |  | 67.8% | 70.1% | 5.70 |
| LVEF < 30% |  | 15.4% | 27.6% | **34.50** |  | 17.6% | 17.0% | 0.97 |  | 16.6% | 19.0% | 6.99 |
| 30-44% |  | 31.5% | 33.7% |  |  | 31.0% | 30.7% |  |  | 31.7% | 32.5% |  |
| ≥ 45% |  | 53.1% | 38.8% |  |  | 51.4% | 51.9% |  |  | 51.7% | 48.9% |  |
| Low systolic BP^1^ |  | 17.8% | 21.5% | 9.21 |  | 17.6% | 16.7% | 1.58 |  | 17.4% | 20.2% | 6.93 |
| Low eGFR^2^ |  | 64.0% | 71.6% | **16.33** |  | 66.5% | 68.5% | 4.24 |  | 65.9% | 64.2% | 3.48 |
| Prolonged QRS duration^3^ |  | 12.5% | 19.2% | **18.50** |  | 13.9% | 16.0% | 5.87 |  | 12.2% | 16.2% | **11.39** |
| Hyponatraemia^4^ |  | 18.0% | 17.9% | 0.26 |  | 18.1% | 17.2% | 2.23 |  | 17.7% | 18.4% | 1.83 |
| Hypokalaemia^5^ |  | 24.4% | 25.4% | 2.31 |  | 24.0% | 26.7% | 6.05 |  | 25.8% | 22.5% | 7.67 |
| Anaemia^6^ |  | 10.2% | 12.0% | 5.60 |  | 10.8% | 11.1% | 1.20 |  | 10.4% | 10.8% | 1.34 |
| Increased BNP/NT-proBNP^7^ |  | 79.9% | 79.9% | 0.18 |  | 79.9% | 78.5% | 3.62 |  | 79.9% | 79.9% | 0.09 |

*Abbreviations.* BNP: B-type natriuretic peptide; BP: blood pressure; COPD: chronic obstructive pulmonary disease; eGFR: glomerular filtration rate; HF: heart failure; LVEF: left ventricular ejection fraction; NT-proBNP: N-terminal pro-B-type natriuretic peptide; SDiff: absolute standardized difference; TIA: transient ischaemic attack.
*Notes.* 1: systolic BP < 115 mmHg; 2: eGFR < 60mL/min/1.73m^2^; 3: QRS > 120 ms; 4: natraemia < 135 mmol/L; 5: kalaemia < 3.8 mmol/L; 6: haemoglobin < 10 g/dL; 7: BNP > 400 pg/mL or (NT-proBNP > 450 pg/mL in patients < 50 years old, NT-proBNP > 900 pg/mL in patients between 50 and 75 years old, NT-proBNP > 1800 pg/mL in patients > 75 years old).

**Table S6.** Characteristics of patients before and after inverse probability of treatment weighting and in preference-based instrumental variable approach in dataset 5.

|  |  | Unweighted population | |  |  | Weighted population | |  |  | Instrumental variable analysis | | |
| --- | --- | --- | --- | --- | --- | --- | --- | --- | --- | --- | --- | --- |
|  | Overall | Control group | HF-DMP group | SDiff % |  | Control group | HF-DMP group | SDiff % |  | No-DMP preference | DMP preference | SDiff % |
| N | 1816 | 1504 | 312 |  |  | 1326 | 297 |  |  | 1141 | 675 |  |
| *Socio-demographic characteristics* | | | | |  |  |  |  |  |  |  |  |
| Male sex |  | 46.9% | 59.6% | **25.61** |  | 48.4% | 49.4% | 2.06 |  | 48.4% | 50.4% | 3.99 |
| Age < 65 years |  | 15.3% | 18.9% | **22.89** |  | 14.2% | 13.8% | 3.35 |  | 15.2% | 17.2% | 8.00 |
| 65-79 years |  | 30.7% | 38.5% |  |  | 31.7% | 30.5% |  |  | 31.4% | 33.2% |  |
| ≥ 80 years |  | 54.0% | 42.6% |  |  | 54.0% | 55.7% |  |  | 53.5% | 49.6% |  |
| Living alone |  | 33.6% | 28.5% | **11.08** |  | 33.0% | 35.4% | 5.14 |  | 32.3% | 33.6% | 2.93 |
| Type of hospital: Local |  | 39.2% | 61.5% | **59.71** |  | 45.8% | 47.3% | **14.95** |  | 43.7% | 42.1% | **137.64** |
| Regional |  | 28.9% | 8.3% |  |  | 22.6% | 16.9% |  |  | 40.4% | 0.0% |  |
| Teaching |  | 31.9% | 30.1% |  |  | 31.6% | 35.8% |  |  | 16.0% | 57.9% |  |
| *Medical history* |  |  |  |  |  |  |  |  |  |  |  |  |
| Ischaemic HF aetiology |  | 21.9% | 32.4% | **23.61** |  | 24.5% | 28.5% | 9.14 |  | 24.4% | 22.7% | 4.00 |
| Diabetes |  | 33.9% | 46.2% | **25.19** |  | 36.6% | 38.4% | 3.77 |  | 34.7% | 38.2% | 7.31 |
| Chronic kidney disease |  | 21.3% | 30.1% | **20.20** |  | 23.5% | 28.0% | **10.18** |  | 22.7% | 23.1% | 0.98 |
| Stroke / TIA |  | 12.7% | 12.8% | 0.36 |  | 12.6% | 10.0% | 8.27 |  | 13.1% | 12.2% | 2.74 |
| COPD / asthma |  | 21.7% | 26.9% | **12.26** |  | 22.8% | 21.5% | 2.98 |  | 23.1% | 21.6% | 3.62 |
| Cancer |  | 15.0% | 11.2% | **11.11** |  | 14.0% | 13.2% | 2.39 |  | 14.6% | 13.9% | 1.78 |
| Previous hospitalisation for acute HF |  | 31.5% | 53.5% | **45.67** |  | 36.7% | 38.1% | 2.84 |  | 35.0% | 35.7% | 1.54 |
| *Clinical and biological status at admission* | | | |  |  |  |  |  |  |  |  |  |
| Overweight or obese |  | 66.4% | 76.3% | **30.21** |  | 67.6% | 66.6% | 5.78 |  | 67.5% | 69.0% | 3.56 |
| LVEF < 30% |  | 16.2% | 26.9% | **32.11** |  | 18.2% | 18.3% | 0.71 |  | 17.8% | 18.5% | 2.75 |
| 30-44% |  | 31.2% | 34.6% |  |  | 31.2% | 31.3% |  |  | 31.6% | 32.2% |  |
| ≥ 45% |  | 52.6% | 38.5% |  |  | 50.7% | 50.4% |  |  | 50.7% | 49.3% |  |
| Low systolic BP^1^ |  | 17.8% | 21.8% | 9.99 |  | 17.7% | 17.1% | 1.61 |  | 17.4% | 20.3% | 7.30 |
| Low eGFR^2^ |  | 64.0% | 71.6% | **16.33** |  | 66.5% | 67.6% | 2.35 |  | 65.9% | 64.2% | 3.48 |
| Prolonged QRS duration^3^ |  | 12.3% | 18.0% | **15.81** |  | 13.6% | 14.3% | 1.83 |  | 12.3% | 15.0% | 7.86 |
| Hyponatraemia^4^ |  | 18.0% | 17.9% | 0.26 |  | 18.2% | 17.8% | 1.08 |  | 17.7% | 18.4% | 1.83 |
| Hypokalaemia^5^ |  | 24.4% | 25.4% | 2.31 |  | 24.0% | 26.7% | 6.33 |  | 25.8% | 22.5% | 7.67 |
| Anaemia^6^ |  | 10.2% | 12.0% | 5.60 |  | 10.8% | 11.0% | 0.56 |  | 10.4% | 10.8% | 1.34 |
| Increased BNP/NT-proBNP^7^ |  | 79.9% | 79.9% | 0.18 |  | 79.9% | 78.9% | 2.59 |  | 79.9% | 79.9% | 0.09 |

*Abbreviations.* BNP: B-type natriuretic peptide; BP: blood pressure; COPD: chronic obstructive pulmonary disease; eGFR: glomerular filtration rate; HF: heart failure; LVEF: left ventricular ejection fraction; NT-proBNP: N-terminal pro-B-type natriuretic peptide; SDiff: absolute standardized difference; TIA: transient ischaemic attack.
*Notes.* 1: systolic BP < 115 mmHg; 2: eGFR < 60mL/min/1.73m^2^; 3: QRS > 120 ms; 4: natraemia < 135 mmol/L; 5: kalaemia < 3.8 mmol/L; 6: haemoglobin < 10 g/dL; 7: BNP > 400 pg/mL or (NT-proBNP > 450 pg/mL in patients < 50 years old, NT-proBNP > 900 pg/mL in patients between 50 and 75 years old, NT-proBNP > 1800 pg/mL in patients > 75 years old).

**Table S7.** Estimations of the heart failure disease management programme effect in the 5 datasets

|  | Dataset 1 | | Dataset 2 | | Dataset 3 | | Dataset 4 | | Dataset 5 | | Pooled results | |
| --- | --- | --- | --- | --- | --- | --- | --- | --- | --- | --- | --- | --- |
|  | HR | 95% IC | HR | 95% IC | HR | 95% IC | HR | 95% IC | HR | 95% IC | HR | 95% IC |
| Model 1: propensity score analysis using IPTW | 0.66 | 0.47 to 0.91 | 0.68 | 0.49 to 0.94 | 0.62 | 0.44 to 0.87 | 0.63 | 0.45 to 0.88 | 0.67 | 0.48 to 0.93 | 0.65 | 0.46 to 0.92 |
| Model 2: model 1 + adjustment for type of hospital and history of chronic kidney disease | 0.64 | 0.46 to 0.89 | 0.66 | 0.48 to 0.92 | 0.61 | 0.43 to 0.85 | 0.61 | 0.44 to 0.85 | 0.65 | 0.47 to 0.90 | 0.63 | 0.45 to 0.89 |
| Model 3: model 1 + trimming of 2.5% of patients on both sides | 0.56 | 0.38 to 0.82 | 0.56 | 0.38 to 0.81 | 0.58 | 0.40 to 0.85 | 0.60 | 0.42 to 0.86 | 0.62 | 0.43 to 0.89 | 0.58 | 0.40 to 0.86 |
| Model 4: model 2 with trimming of 2.5% of patients on both sides | 0.55 | 0.38 to .81 | 0.55 | 0.38 to 0.80 | 0.58 | 0.40 to 0.84 | 0.60 | 0.41 to 0.86 | 0.61 | 0.43 to 0.88 | 0.58 | 0.39 to 0.85 |
| Instrumental variable | 0.58 | 0.28 to 1.20 | 0.58 | 0.28 to 1.20 | 0.56 | 0.27 to 1.14 | 0.55 | 0.27 to 1.14 | 0.53 | 0.25 to 1.09 | 0.56 | 0.27 to 1.16 |

*Abbreviations.* 95% CI: 95% confidence interval; HR: hazard ratio; IPTW: Inverse probability of treatment weighting.
